# Cell Observation and Analysis with a Three-Dimensional Optical Wave Field Microscope

**DOI:** 10.3390/bios15080515

**Published:** 2025-08-08

**Authors:** Shimon Matsumoto, Shoko Itakura, Junta Minato, Masahiro Hashimoto, Shu Obana, Mai Kanai, Masaki Kobayashi, Makiya Nishikawa, Kosuke Kusamori

**Affiliations:** 1 Otsuka Electronics, 1-10 Sasagaoka, Koka 528-0061, Japan; matsumoto.shimon@otsuka.jp; 2 Laboratory of Biopharmaceutics, Faculty of Pharmaceutical Sciences, Tokyo University of Science, 6-3-1 Niijuku, Katsushika, Tokyo 125-8585, Japan; itakura@rs.tus.ac.jp (S.I.); 3b24541@ed.tus.ac.jp (M.H.); 3a22703@ed.tus.ac.jp (M.K.); makiya@rs.tus.ac.jp (M.N.); 3 Laboratory of Cellular Drug Discovery and Development, Faculty of Pharmaceutical Sciences, Tokyo University of Science, 6-3-1 Niijuku, Katsushika, Tokyo 125-8585, Japan; 3b24549@ed.tus.ac.jp (J.M.); 3a24704@ed.tus.ac.jp (S.O.); 4 Department of Food and Nutrition Science, Graduate School of Humanities and Sciences, Ochanomizu University, 2-1-1 Otsuka, Bunkyo, Tokyo 112-8610, Japan; kobayashi.masaki2@ocha.ac.jp; 5 Institute for Human Life Science, Ochanomizu University, 2-1-1 Otsuka, Bunkyo, Tokyo 112-8610, Japan

**Keywords:** 3D-OWFM, cell observation, imaging, label-free, microscopy, optical wave

## Abstract

Cell observation is crucial in life science research, and advancements in microscopy are essential for deciphering biological phenomena. These technological developments have significantly enhanced our understanding of cellular mechanisms and processes. Light, characterized by its wave-like properties, is fundamental to scientific observation. Recently, new technologies have been developed to detect changes in light wavelengths upon illumination, using them as signals for visualization. Three-dimensional optical wave field microscopy (3D-OWFM), a recent innovation in optimal imaging, leverages the wave properties of light to capture objects without labels, invasive procedures, or direct contact, thus facilitating non-invasive observation. In this study, we observed and analyzed mammalian cell structure and behaviors using 3D-OWFM. The 3D-OWFM revealed the intrinsic structure of the cells, including the cytoplasm and nucleus, with high clarity. The optical path difference (OPD) intensity effectively highlighted nuclear complexity. Furthermore, time-lapse imaging captured cell division process through variations in OPD signal intensity. These findings indicate that 3D-OWFM has significant potential for cell observation, offering insights not attainable with conventional microscopes.

## 1. Introduction

Cells are the fundamental units of life that provide structural and functional organization to all living organisms [1]. Their intricate mechanisms underlie essential biological processes, from development to environmental adaptation [2]. Cellular research is crucial for deciphering the complexities of life because it allows us to explore the underlying mechanisms of living systems [3]. Recent advances in single-cell analysis techniques have deepened our knowledge of cellular heterogeneity, highlighting the importance of innovative tools in cellular research [4,5]. With improvements in microscopic technology, researchers can reveal previously unknown biological processes and provide deeper insights into cellular dynamics [6]. These advances have significantly enhanced our understanding of the relationships between cellular behavior and various physiological and pathological conditions [7]. Phase-contrast microscopy, a general microscopic technique, allows for visualization of transparent specimens without staining, making it ideal for living-cell imaging [8]. Confocal laser scanning microscopy provides high-resolution, three-dimensional imaging by scanning a specimen with a laser, which is particularly useful for detailed studies of cellular structures [9]. This technique is widely used in live-cell imaging because of its ability to minimize out-of-focus light and enhance contrast [10]. Scanning electron microscopy (SEM) provides high-magnification surface-level imaging, revealing the fine details of the cell surface [11]. Transmission electron microscopy (TEM) enables the study of intracellular components at the nanoscale by passing electrons through thin specimens [12]. However, phase-contrast microscopy may lack fine details in complex samples, and confocal laser scanning microscopy may be limited by longer imaging times and potential photodamage to the samples. SEM and TEM also require complicated sample preparation and are typically limited to nonliving samples. Therefore, the development of more advanced microscopes continues to overcome these issues and improve our ability to study cellular structures.

Light as an electromagnetic wave has been used in various techniques to analyze the fine structure of materials without direct contact [13]. Holographic microscopy is one of many methods that exploit the phase and amplitude of light to achieve high-contrast, label-free imaging of living cells [14]. Quantitative phase imaging (QPI) extends these capabilities by providing quantitative information on cellular morphology and dynamics [13,15]. These techniques underscore the versatility of light-based imaging for studying cellular structures without requiring physical contact. Recent developments, such as digital holography and optical diffraction tomography, demonstrate the ability of optical methods to capture cellular behavior in real time with submicron precision [14,16]. Among these, three-dimensional optical wave field microscopy (3D-OWFM) represents a groundbreaking development. By analyzing the optical wave field using wave equations, 3D-OWFM enables label-free, non-invasive, and real-time observation of internal structures. Similar principles were applied in practical scenarios, including monitoring cellular responses to environmental changes using digital holography and assessing refractive index distributions in cells using optical diffraction tomography [17]. These methods highlight the potential of 3D-OWFM to revolutionize cellular imaging in fields ranging from biology to nanotechnology. However, living cells were not observed using the 3D-OWFM. To the best of our knowledge, this is the first study to apply 3D-OWFM to unstained living cells. Unlike conventional QPI techniques, our system captures both phase and amplitude information simultaneously, providing enhanced flexibility for wavefield reconstruction.

In this study, we developed a novel 3D-OWFM system, which was utilized to observe and analyze the structure of mammalian cells. First, we observed mesenchymal stromal cells and hepatoblastoma cells using the optical path difference (OPD) signal and then analyzed their structure. We then processed the OPD signals obtained from the cells to generate two types of visual outputs: OPD edge images, which emphasize the cellular boundaries, and OPD differential images, which provide a three-dimensional perspective of the observed structures. Finally, we performed time-lapse observations of the cells and analyzed cell division based on the OPD signal changes.

## 2. Materials and Methods

### 2.1. System of 3D-OWFM

**Figure 1:**
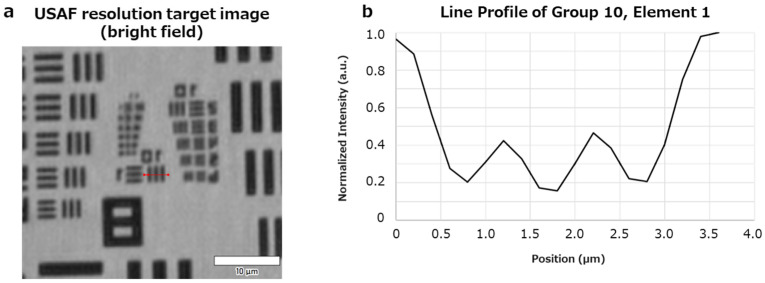
Resolution evaluation using a USAF 1951 resolution test target. (**a**) Bright-field image acquired by MINUK showing the USAF test pattern. The red line indicates the position along which the cross-sectional intensity was measured. (**b**) Intensity profile along the red line in (**a**), corresponding to Group 10, Element 1. Distinct intensity variations indicate successful resolution of fine structures. Scale bar: 10 μm.

The 3D-OWFM used in this study consisted of an optical system with a laser light source of 638 nm wavelength, which has high coherence essential for accurate OPD, and a wave sensor (Otsuka Electronics, Osaka, Japan), along with dedicated imaging software (Otsuka Electronics). The sample was irradiated with laser light, and the transmitted light was detected using a wave sensor. The light wave information obtained by this sensor was transmitted in real time to a computer within the system for analysis. The interference pattern detected by the wave sensor is numerically analyzed using the wave equation to extract the OPD distribution, which is then visualized as a high-contrast image. To evaluate the spatial resolution of the system, we used a USAF 1951 resolution test target and analyzed the optical wave information acquired by the system. As shown in Figure 1, the system clearly resolved Group 10, Element 1, which corresponds to a lateral resolution of approximately 0.49 μm. While switching to near-infrared (NIR) light would reduce the spatial resolution due to the longer wavelength, it would enhance light penetration into the sample and reduce scattering and reflection, thereby improving the visibility of internal structures. The OPD is a physical quantity proportional to the thickness and refractive index difference in the sample and is calculated as follows:

$$OPD\;=\;(n_{2}\;-\;n_{1})d$$

where $n_{1}$ is the refractive index of the surrounding medium, $n_{2}$ is the refractive index of the region of interest (e.g., cells), and d is the thickness of the sample. Therefore, OPD images reflect not only the planar morphology (x − y) but also z-directional information based on variations in refractive index and thickness. This process enables the visualization of detailed internal structures, even in unstained cells, based on refractive-index variations. In 3D-OWFM, the fine structure of an object is visualized by analyzing the transmitted laser light. The wave sensor used in the 3D-OWFM system acquires optical wave information by off-axis holography. It captures interference fringes formed by the object and reference waves, which are recorded on the sensor surface. To ensure accurate reconstruction, a calibration sample with a known structure is first used to adjust the reference beam parameters. The recorded hologram is then numerically processed to extract both amplitude and phase components of the wavefront. The phase information is converted into an OPD image, which reflects variations in refractive index and sample thickness, allowing for high-fidelity, label-free imaging of internal cellular structures. To characterize the system’s imaging performance, we evaluated both the field of view (FOV) and the lateral resolution. The FOV of the 3D-OWFM system is 700 μm × 700 μm, which is governed by the pixel pitch and the total number of pixels in the reconstructed image. These parameters also define the lateral resolution limit of the system. To quantify the resolution, a USAF 1951 resolution test target was used. As shown in Figure 1, Group 10, Element 1 was clearly resolved, corresponding to a lateral resolution of approximately 0.49 μm.

### 2.2. Convert of OPD Images to OPD Edge and OPD Differential Images

In this study, OPD images, OPD edge images, and OPD differential images obtained using the 3D-OWFM were used for analysis. The OPD images were computed by analyzing the optical wave information from the sample to determine the OPD. The OPD image is calculated based on the phase difference between the optical wave distribution from the sample and that of the illumination light. The optical wave distribution of the sample is expressed as A(x,y), and that of the illumination light is expressed as Q(x,y). The distributions are as follows:

$$A(x,y)=A_{0}(x,y)exp(i\varphi_{A})$$

$$Q(x,y)=R_{0}(x,y)exp(i\varphi_{R})$$

where $A_{0}(x,y)$ and $R_{0}(x,y)$ are the amplitude components, and $\varphi_{A}(x,y)$ and $\varphi_{R}(x,y)$ are the phase components. Therefore, an OPD image is defined as follows:

$$OPD(x,y)=\frac{\lambda }{2\pi }[\varphi_{A}(x,y)-\varphi_{R}(x,y)]$$

where λ represents the wavelength of the light used for observation. By converting the obtained OPD images, OPD edge and differential images can be generated. The OPD edge image is generated by computing the gradient of the OPD image. Edge intensity E(x,y) was determined using the following equation:

$$E\left(x,y\right)=\sqrt{{\left(\frac{\partial A}{\partial x}\right)}^{2}+{\left(\frac{\partial A}{\partial y}\right)}^{2}}$$

Through this process, local variations in the OPD image were emphasized to extract contour information.

However, the OPD differential image was generated by calculating the directional derivative of the OPD image. The absolute value of the directional derivative of the OPD along the unit vector r, which represents the differentiation direction, is given as follows:

$$D(x,y)\;=\frac{\partial OPD}{\partial r}$$

This process extracts the amount of OPD variation in a specified direction. In this study, these images were generated using 3D-OWFM analysis functions and were subsequently utilized for the morphological and dynamic analysis of the cells.

### 2.3. Analysis Software

Data acquisition and analysis for the 3D-OWFM system were supported by two dedicated software tools: MINUK (version 2.7.0.0) and MINUK Viewer (version 2.13.2.0). These software tools are designed to facilitate real-time data capture and post-acquisition analysis. The capture software, MINUK, is responsible for controlling the hardware, capturing data, and saving data in standard formats. The user interface of MINUK is divided into three main sections: toolbar, reconstructed image display area, and side panel. The software automatically initiates the capture mode once hardware initialization is complete, ensuring seamless operation. During data acquisition, users can save optical wave data in standard file formats such as *. shi for the standard wave field data, and *. sci for the complex image data. These files served as the foundation for further analyses. For post-acquisition analysis, the dedicated viewing software MINUK Viewer was used. This software allows users to open and analyze saved data files, providing functionality similar to the capture software, but with some hardware-related features, such as stage movement and disability. The MINUK Viewer supports the simultaneous opening of multiple instances and provides sufficient system memory. Users can visualize and process the standard wavefield data (.shi) and complex image data (.sci) by dragging and dropping files into the application, selecting them through the toolbar, or double-clicking files directly from the file explorer. Both software tools are equipped with features for visualizing OPD, edge, and differential images. In addition, ImageJ (version 1.54g), a public domain Java-based image analysis program developed by the National Institutes of Health [18], was used for the pseudo-3D reconstruction.

### 2.4. Cell Culture and Observation Condition

C3H10T1/2 cells, kindly provided by Dr. Hiroki Kagawa (Kyoto, Japan), were cultured in DMEM (Nacalai Tesque Inc., Kyoto, Japan) supplemented with 15% heat-inactivated fetal bovine serum (FBS). NIH3T3 cells, purchased from RIKEN Bioresource Center (RIKEN BRC) (Tsukuba, Japan), were cultured in DMEM (high glucose) supplemented with 10% heat-inactivated FBS. HaCaT cells, purchased from Cell Line Service (Eppelheim, Germany), were cultured in DMEM (high glucose) supplemented with 10% heat-inactivated FBS. IMS32 cells, purchased from Cosmo Bio Co., Ltd., Sapporo Office (Sapporo, Japan), were cultured in Schwann Cell (IMS32) Medium (Cosmo Bio Co., Tokyo, Japan). RAW264.7 cells, purchased from Sumisho Pharma (Tokyo, Japan), were cultured in RPMI 1640 (Nacalai Tesque Inc.) supplemented with 10% heat-inactivated FBS. All cells were maintained in a humidified atmosphere containing 5% CO_2_ at 37 °C.

### 2.5. Time-Lapse Imaging and Cell Survival Assay

Time-lapse images of C3H10T1/2 cells were taken at 30 s intervals for a total of 3 h using 3D-OWFM. The cells were cultured in an incubation chamber (SV-70A; BLAST Co., Kawasaki, Japan) that maintained a humidified atmosphere containing 5% CO_2_ at 37 °C condition during time-lapse imaging. To evaluate cell survival, C3H10T1/2 cells were seeded in a 35 mm culture dish at a density of 5 × 10^4^ cells, and time-lapse imaging was performed. Cell numbers were measured using the Cell Counting Kit-8 (CCK-8) (Dojindo Laboratories, Kumamoto, Japan) after 0, 1, 3, 6, and 24 h of incubation. For the analysis of dead cells during time-lapse imaging, cells were collected using trypsin-ethylenediaminetetraacetic acid (EDTA) solution (Nacalai Tesque Inc.) after 24 h of incubation. The cells were stained with Annexin V-633 Apoptosis Detection Kit (Nacalai Tesque Inc.) and analyzed using a BD FACSLyric (Becton Dickinson, San Jose, CA, USA). The proportion of apoptotic cells was calculated using FlowJo software version 10.7.2 (Becton Dickinson).

### 2.6. Statistical Analysis

Statistical differences were evaluated using one-way analysis of variance (ANOVA), followed by Student’s *t*-test for comparisons between two groups. Statistical significance was set at *p* < 0.05.

### 2.7. Use of Generative Artificial Intelligence Tools

Generative artificial intelligence (GenAI) tools, specifically ChatGPT (GPT-4, OpenAI, San Francisco, CA, USA), were used to assist in drafting and refining English expressions. All content was reviewed and finalized by the authors and underwent professional English editing by an external proofreading service.

## 3. Results

### 3.1. Appearance of the 3D-OWFM System and the Visualization Principles

**Figure 2:**
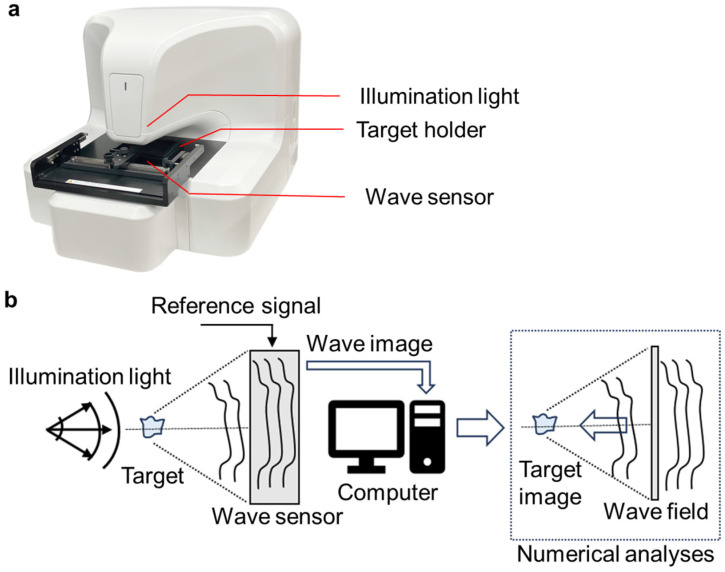
(**a**) Appearance of 3D-OWFM. (**b**) Equipment of 3D-OWFM. The optical wave is radiated from a laser light source, emitting an optical wave at a wavelength of 638 nm, which is directed through an optical pathway to uniformly illuminate the sample from the top of the device. As the light passes through the sample within the sample holder, it interacts with the cellular structures, thereby causing phase shifts in the optical wave. These phase shifts result from differences in refractive index and thickness within the sample. The wave sensor located beneath the sample detects these phase shifts and processes them into OPD signals, which reflect the structural variations in the sample. The OPD represents the difference in optical path lengths between a specific point and reference point within the sample. This occurs because light propagating through a medium with a refractive index has a wavelength reduced to λ/n, causing variations in optical distance even if the physical distance remains constant. By mapping the spatial distribution of these optical path lengths, the OPD distribution can be visualized, enabling precise analysis of the sample’s internal structure. This process allows the 3D-OWFM to generate high-contrast, label-free images of cellular structures, revealing details that are otherwise difficult to observe through conventional microscopy.

**Figure 3:**
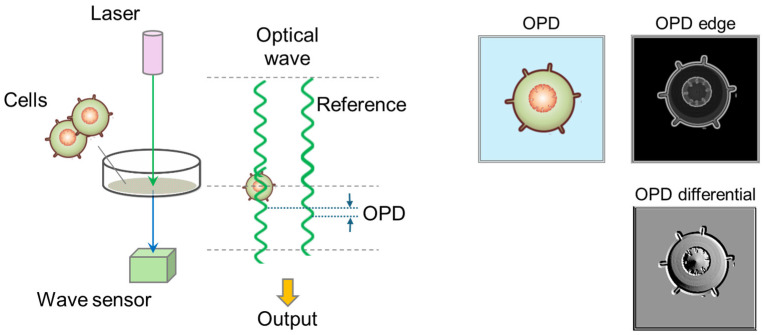
Visualization principle of 3D-OWFM. OPD signal is converted from the wave signal to visualize the observed cells. OPD edge visualizes the edges of the OPD image weighted by light amplitude, enabling high-contrast visualization of transparent samples compared to conventional edge detection methods. OPD differential calculates the derivative of the OPD in a specified direction, generating a pseudo-relief image with a sense of depth. By changing the differentiation direction, it becomes easier to identify directional striated structures within the sample.

Figure 2a,b show the overall image of the 3D-OWFM system and its details, respectively. The 3D-OWFM system comprises three components: a laser light source, a wave sensor, and specialized imaging software. A laser with a wavelength of 638 nm was used as the illumination light, which was irradiated from the top of the device. In this system, the laser beam is neither focused onto the sample nor strictly collimated. Instead, a laser beam with a divergence angle of approximately 14 degrees is used to uniformly illuminate the entire field of view (700 μm × 700 μm). The light transmitted from the sample was detected by a wave sensor located at the bottom of the device. This system uses Fourier transform optics to eliminate conjugate images. The detected optical wave includes the optical wave distribution of the sample and the known reference wavefront, allowing for the exact optical wave distribution of the object. These distributions were acquired as wave images, which were transmitted to a computer for analysis. Numerical analyses were performed on these wave images to reconstruct the wave field and generate a target image. Through this process, the detailed refractive-index distribution and structural information of the sample were visualized with high precision. In addition, using a lensless configuration, the system captures the 0.6 μm wavefront in the xy-plane and aberration-free OPD imaging with nanometer resolution in the z-direction. Supplementary Figure S1 shows images of C3H10T1/2 cells using conventional in-line holography and 3D-OWFM. With conventional in-line holography, conjugate image artifacts and other interference effects often degraded image clarity, making it difficult to accurately visualize the morphology of transparent cells. In contrast, 3D-OWFM provided OPD images that accurately represented cell shape, demonstrating the system’s strength in capturing transparent biological structures. To further evaluate the system’s ability to visualize three-dimensional structure, we conducted additional experiments using two types of cells. In Supplementary Figure S2a, an OPD image of C3H10T1/2 cells was used to extract a region of interest, from which a 3D shape was generated. This clearly demonstrated structural variation along the z-direction. In Supplementary Figure S2b, we acquired z-stacked OPD images of RAW264.7 cells at 1 μm intervals from +10 μm to −20 μm relative to the cell surface. These images were obtained from volumetric wave information acquired in a single capture across a ±700 μm focal depth range. A pseudo-3D image was reconstructed from this dataset, effectively visualizing structural changes along the z-axis. The system was equipped with a motorized XY stage and a manual XY stage to precisely position the sample. The motorized stage enables movements of ±10 mm in both the X and Y directions, while the manual stage offers a similar range of motion for fine adjustments and a travel range of 129 mm in the X direction and 85 mm in the Y direction. A target holder was attached to the stage, enabling the measurement of samples up to 100 mm × 80 mm × 20 mm in size. These features ensure compatibility with various experimental setups, including culture dishes and multiwell plates. Figure 3 shows the visualization principle of the 3D-OWFM system. Laser light was directed onto the sample through an optical pathway designed to provide uniform illumination. To enhance the image quality and minimize artifacts, multiple illumination conditions were used, and the recorded data were processed to improve the focus selection and signal-to-noise ratio. The radiated light wave interacts with the components of the adhered cells on the culture dish, causing changes in the phase of the optical wave. These changes were proportional to the refractive index and thickness of the cellular structures. The modulated optical wave distribution from the sample was then detected using a wave sensor, and the difference from the reference signal was measured as the OPD, thereby generating data corresponding to the spatial distribution of the refractive indices and heights. The measured OPD data were processed using specialized imaging software that enabled high-contrast visualization. The processed OPD data can be visualized in two complementary ways: OPD edge images, which highlight gradients in the OPD distribution and emphasize cellular boundaries and fine structural details, allowing for clear observation of cell boundaries and fine structures without being affected by halo artifacts commonly seen in phase-contrast microscopy. OPD differential images, which provide pseudo-relief representations to enhance the visualization of depth and directional features within the sample, contributing to three-dimensional morphological analysis. These imaging modes are useful for analyzing transparent cellular structures.

### 3.2. Observation and Analysis of Cells with 3D-OWFM

**Figure 4:**
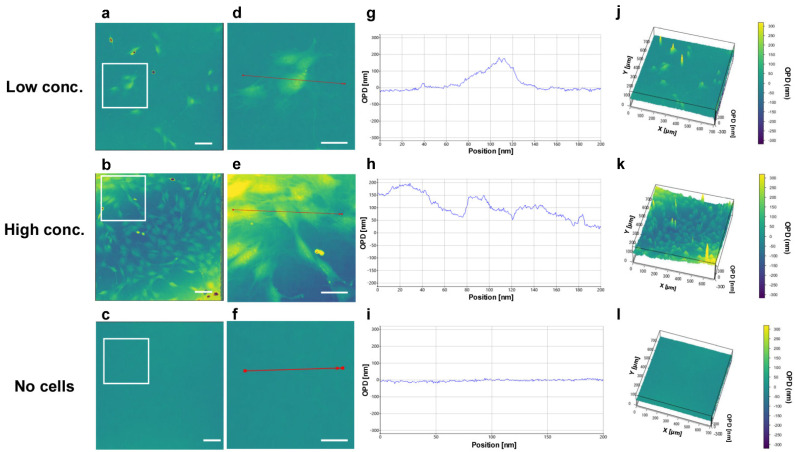
Observation of cells with 3D-OWFM. C3H10T1/2 cells at low cell density (**a**,**d**,**g**,**j**) and high cell density (**b**,**e**,**h**,**k**), and medium without cells (**c**,**f**,**i**,**l**) were observed using 3D-OWFM. White boxes in panels (**a**–**c**) indicate the regions of interest (ROIs) shown enlarged in panels (**d**–**f**). Red lines in panels (**d**–**f**) indicate the positions along which the cross-sectional OPD profiles shown in panels (**g**–**i**) were measured. The OPD signal intensity was approximately 200 nm under low-cell-density conditions, ranging from approximately 20 nm to 200 nm under high-cell-density conditions, and showed little variation in medium without cells. The scale bars indicate 100 μm.

Figure 4 shows OPD images of the mouse mesenchymal stromal cell line C3H10T1/2 at varying cell densities. The cell structure and cell boundaries of C3H10T1/2 cells at both densities were clearly observed, whereas no signals were detected in the no-cell group (Figure 4a–c). Figure 4d–f show enlarged images of Figure 4a–c. Figure 4g–i show the OPD signal intensity in the indicated linear range (red lines in Figure 4d–f), indicating that the OPD signal intensity reflects the cell structure and height. The OPD value corresponds to the optical path difference, calculated as $OPD=(n_{2}-n_{1})d$, where $n_{1}$ and $n_{2}$ are the refractive indices of the surrounding medium (e.g., culture medium) and the cells, respectively, and d represents the cell thickness. These measurements enabled the precise analysis of the cell structure and height. The OPD values obtained in this study represent the minimum path difference constrained by the 2π phase wrapping effect. For instance, with an estimated refractive index difference of 0.02, a measured OPD of 200 nm would correspond to an actual sample thickness of approximately 10 μm. Although phase unwrapping was not applied in the present analysis, the resolution achieved is sufficient for qualitative interpretation of structural differences. In addition, the cell structure was rendered in three dimensions based on the OPD signal intensity (Figure 4j–l).

### 3.3. Observation of Various Cells with 3D-OWFM and Comparison to Digital Microscope

**Figure 5:**
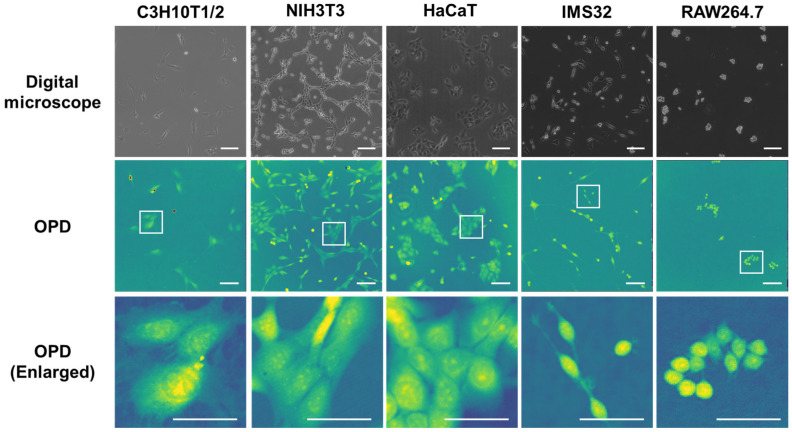
OPD images of various cells observed using 3D-OWFM. C3H10T1/2 cells, HepG2 cells, HaCaT cells, IMS 32 cells, and RAW264.7 cells were observed using a digital microscope and 3D-OWFM. White boxes in the digital microscope images indicate the regions of interest (ROIs) shown enlarged in the corresponding 3D-OWFM OPD images. Enlarged images of various cells observed using 3D-OWFM. The OPD signal intensity ranged from approximately 20 nm to 200 nm. The scale bars indicate 100 μm (OPD, OPD enlarged) and 50 μm (Digital microscope).

**Figure 6:**
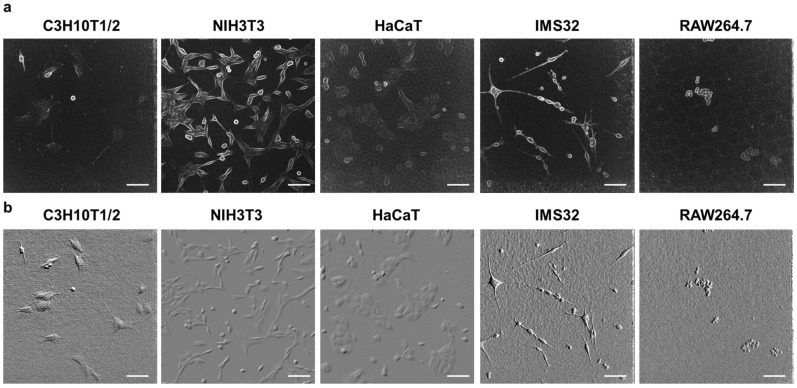
OPD edge and OPD differential images of various cells observed using 3D-OWFM. OPD images of C3H10T1/2 cells, HepG2 cells, HaCaT cells, IMS 32 cells, and RAW264.7 cells were converted to OPD edge (**a**) and OPD differential images (**b**). The OPD signal intensity ranged from approximately 20 nm to 200 nm. The scale bars indicate 100 μm. For OPD differential images in panel (**b**), the OPD map was differentiated along a specified direction in the xy-plane for each cell type. The differentiation directions were as follows (in degrees): C3H10T1/2—115°; NIH3T3—45°; HaCaT—45°; IMS32—179°; and RAW264.7—179°.

Then, five different types of mammalian cells were observed, and similar signal information was obtained for each. When the data collected from the cells were compared, differences in cell size, shape, and the size and shape of the angles were evident, and these features were highlighted by the OPD signals. Additionally, in C3H10T1/2 cells, tube-like structures similar to tunneling nanotubes were observed. In IMS32 cells, elongated protrusions were also observed. Figure 5 shows images of C3H10T1/2 cells, mouse fibroblast cell line NIH3T3 cells, human keratinocyte cell line HaCaT cells, mouse Schwann cell line IMS 32 cells, and mouse macrophage-like cell line RAW264.7 cells observed using two distinct methods: a digital microscope and 3D-OWFM. The cells have their own size and shape. The OPD image showed the same size and shape as observed with a digital microscope, indicating that it is possible to observe the whole shape of the cell. In addition, the enlarged images from the OPD provided detailed information about the internal structure of the cells, especially of the nucleus, which is difficult to observe with a digital microscope. Furthermore, the analysis of the enlarged OPD images revealed more detailed intracellular structures. In particular, aggregates in the nucleus were observed in the cells under study. In contrast, the confocal laser scanning microscope image shows that the outline of the nucleus can be seen, but the internal structure of the nucleus cannot be observed without nuclear staining (Supplementary Figure S3). Figure 6a,b show the OPD edge and differential images of various cells, respectively. The OPD images were converted into OPD edges or differential images. The OPD edge images of the respective cells emphasized the gradient of the OPD distribution, enabling high-contrast visualization of transparent samples where significant changes in the OPD occurred. The OPD differential images of the respective cells were pseudo-relief images obtained by differentiating the OPD in a specified direction, making it easier to recognize the directional striated structures. OPD differential images were computed by differentiating the OPD map along the xy-plane.

### 3.4. Time-Lapse Observation of C3H10T1/2 Cells Using 3D-OWFM for the Analysis of Cell Division

**Figure 7:**
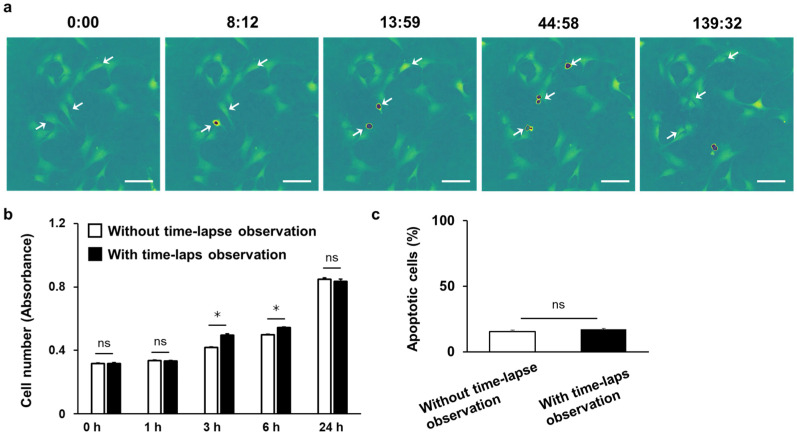
Time-lapse observation of cells using 3D-OWFM. (**a**) Time-lapse images of C3H10T1/2 cells using 3D-OWFM were captured from the time-lapse movie (Video S1). The numbers above images indicate minutes and seconds. The OPD signal intensity ranged from approximately 20 nm to 200 nm. The scale bars indicate 100 μm. White arrows indicate nuclei showing OPD signals during cell division. (**b**) Cell number and (**c**) apoptotic ratio of C3H10T1/2 cells 24 h after time-lapse observation. Results are expressed as mean ± SD of three samples. * *p* < 0.05 versus without time-lapse observation. ns: not significant.

Figure 7a shows the time-lapse OPD images of C3H10T1/2 cells acquired using the 3D-OWFM from a time-lapse movie (Video S1). As indicated by white arrows, the nuclei of several cells exhibited significantly strong OPD signals during cell division (Figure 7a). Upon division into two cells, OPD signals in the nuclei decreased to a level equivalent to that observed before division. In addition, because the cell number and ratio of apoptotic cells remained equivalent to the control condition (without the time-lapse observation group) within 24 h of incubation (Figure 7b,c), long-term observation with the 3D-OWFM had minimal effects on cell viability.

## 4. Discussion

### 4.1. Technical Advantages of 3D-OWFM

Recent advances in microscopy have led to the development of innovative techniques for cell observation. Among these, 3D-OWFM has been developed primarily for visualizing transparent industrial materials. In the present study, we demonstrated for the first time that this system is also applicable to live-cell imaging, enabling non-invasive acquisition of morphological and structural information using OPD images. These findings extend the applicability of 3D-OWFM to biological contexts and provide a foundation for its further use in cellular studies. These include flow cytometry-integrated microscopy [19], which facilitates concurrent analysis of cell populations and their imaging; Raman spectroscopy-based microscopy for chemical composition analysis at the single-cell level [20]; and holographic microscopy [21], which enables label-free, three-dimensional imaging of living cells. Each technology offers unique advantages, such as high specificity, quantitative analysis, and non-invasive imaging, rendering them suitable for different applications. Compared to these microscopy techniques, 3D-OWFM offers several advantages. Although OPD is defined as the product of the refractive index and sample thickness, the 3D-OWFM system cannot independently resolve these two parameters. Nevertheless, the OPD distribution itself provides valuable three-dimensional structural information. Specifically, the OPD images obtained with the system reflect the integrated optical properties along the depth direction. Furthermore, numerical refocusing after acquisition enables pseudo-three-dimensional visualization of focal planes at different depths, allowing for qualitative evaluation of spatial structures and relative height differences within biological samples. Conventional optical microscopy [8,9] requires fluorescent labeling to observe cell structures such as nuclei and involves high preparation costs and time, whereas lensless holographic microscopy [22,23,24] enables label-free imaging, preserving the natural state of cells. This eliminates potential artifacts or damage caused by foreign substances, making it particularly advantageous for live-cell imaging. Another advantage is the ability to observe cell structures without glass-based dishes or high-power lenses, thereby facilitating streamlined processes. The system is also highly efficient, requires minimal preparation, and allows for immediate observation. Its ability to perform real-time OPD imaging allows for the simultaneous detection of complex cellular information, such as morphological changes and refractive index variations, without damaging the cells. By avoiding the need for labeling or staining, phase-based imaging techniques enable continuous, non-invasive observation of living cells over time, including particle transport and intracellular dynamics, morphological changes, the movement of suspended cells, and changes in cell density or dry mass. Additionally, 3D-OWFM is similar to lensless holographic microscopes at these points but it can be clearly distinguished from those studies in two key aspects: (1) it uses a known reference wavefront, allowing for accurate acquisition of the optical wave from the sample without introducing aberrations; and (2) the system eliminates conjugate images, enabling precise measurement of OPD. Therefore, 3D-OWFM allows it to avoid aberrations common in conventional microscopy and enables precise, quantitative observations (Supplementary Figure S1). The 3D-OWFM system is particularly advantageous for studying cell dynamics and interactions because it combines several features—label-free, time-lapse imaging, and no requirement for manual focus adjustment. These features are suitable for routine applications and make them easy to use, while providing detailed structural and functional information. In contrast, 3D-OWFM offers fast and accurate structural information, complementing other advanced microscopic techniques by enabling real-time, non-invasive observations. As a result, the 3D-OWFM is considered a highly versatile and multifunctional microscope. This provides a new way to observe dynamic cellular processes with high precision and minimal intervention. These characteristics make it particularly useful for a wide range of biological research, including live-cell imaging, tissue engineering, and drug development. In addition, compared to other three-dimensional imaging techniques such as optical coherence tomography (OCT), which generally provide axial resolution in the micrometer range, the 3D-OWFM system achieves nanometer-scale resolution in the z direction, approximately 10 nm, and submicron resolution in the xy plane, approximately 0.6 μm. Furthermore, while conventional holographic microscopy typically offers limited focus selectivity, 3D-OWFM enables focal plane selection with intervals as fine as 1 μm. These characteristics suggest that the 3D-OWFM system is advantageous for detailed morphological analysis requiring precise z-directional information.

### 4.2. Interpretation of Key Findings

The OPD signal, detected by changes in the refractive index, provides information on the cell height to be extracted (Figure 4). It has previously been shown that in uniform materials such as thin polystyrene films, the refractive index can be used to accurately calculate the thickness [25]. However, this study revealed that the cell nuclei exhibited high OPD signal intensities (Figure 4), with exceptionally strong signals observed during cell division (Figure 7a). Although these prominent signals interfere with the accurate measurement of the cell height, they provide cell-specific information. Notably, the ability of OPD imaging to visualize nuclear structures allows for real-time observation of dynamic changes during mitosis. As cell division proceeds, the nuclear structure undergoes dynamic changes. During mitosis, the nuclear envelope disassembles, allowing chromosomes to segregate. The nuclear envelope then reassembles to restore nuclear architecture [26]. The strong OPD signals corresponding to these structural transitions highlight the technique’s sensitivity to mitotic events. High-resolution imaging revealed detailed intracellular features including nuclear aggregates (Figure 5), whereas confocal imaging cannot observe the internal structure of the nucleus without staining (Supplementary Figure S3), highlighting the ability of OPD imaging to capture intricate cellular components. These findings suggest that OPD imaging holds significant potential for real-time assessment of cellular functions, particularly through the analysis of nuclear dynamics. The observation of cell structures using OPD signals requires further investigation, and the detailed observation of intracellular structures is likely to become possible with the advancement of analytical methods [27].

The 3D-OWFM used in this study was equipped with a 638 nm laser (Figure 3), no cytotoxicity was observed during the 24 h time-lapse (Figure 7b,c), and the cellular structures were observed in detail. The wavelength of the laser is freely selectable, and by adjusting it to near-infrared (NIR) wavelengths, such as 830 nm or 1030 nm, improved imaging results can be expected. Specifically, NIR wavelengths can reduce surface reflections and internal scattering, allowing for clearer visualization of both cell surfaces and internal structures. This capability makes the 3D-OWFM system highly adaptable to different experimental conditions, particularly for thick or highly scattered biological samples [24]. The laser illumination mode also significantly affected the quality of the observed images. The 3D-OWFM system offers several illumination settings, including wide-angle, standard-angle, and narrow-angle modes, each suited to a specific observation requirement. Wide-angle illumination minimizes the effect of defocused light, making it particularly effective for observing specimens with minimal height variations. However, narrow-angle illumination provides enhanced focus on high-relief structures, improving contrast and resolution of images with significant height differences. These adjustable illumination modes allow researchers to optimize the system for a wide range of biological samples. By offering tailored imaging solutions for different sample types and experimental scenarios, the 3D-OWFM is a powerful tool for advancing research in cell biology and beyond.

Traditional microscopes struggle to capture such detailed cellular information, demonstrating the utility of 3D-OWFM and suggesting promising future applications.

The 3D-OWFM system has significant potential for applications beyond cellular imaging, owing to its ability to detect subtle changes in the refractive index. This capability is particularly promising for identifying structural changes during disease progression, such as cancer metastasis or fibrosis, in which refractive index variations are key indicators of pathological states. By analyzing refractive properties and morphological features, it may be possible to establish new evaluation parameters and detect early signs of malignancy. These potential applications suggest that 3D-OWFM can contribute to advances in biomedical research and diagnostics. Future software updates incorporating machine learning or automated pattern recognition could further enhance its capabilities, allowing for automated detection of specific cellular or material features. Moreover, the flexibility of the 3D-OWFM system in adjusting the laser wavelengths and imaging modes provides a basis for innovative research in multidisciplinary fields. For instance, optimizing the system for near-infrared wavelengths could facilitate studies of opaque or highly scattered samples, such as tissues or composites. These attributes make the 3D-OWFM a versatile research tool that combines imaging precision, adaptability, and ease of use, making it suitable for a wide range of applications. Furthermore, in the field of drug discovery, 3D-OWFM can serve as a powerful platform for high-throughput screening by assessing the effects of pharmacological agents on living cells. By integrating its unique capabilities into both biological and material science applications, the 3D-OWFM system is expected to revolutionize the way researchers tackle complex imaging challenges and pave the way for new scientific and technological discoveries.

### 4.3. Future Applications

The ability of 3D-OWFM to detect subtle morphological and refractive-index variations suggests promising applications beyond standard cell imaging. For example, based on the distinct optical information obtained from various cell types, this system could be adapted for detecting different cell populations and analyzing intercellular interactions in co-cultures of multiple cell types. Furthermore, its non-invasive, label-free imaging capability may be leveraged in drug development, tissue engineering, and real-time monitoring of cellular dynamics. These features position 3D-OWFM as a powerful platform for both fundamental research and applied biomedical studies.

## 5. Conclusions

The 3D-OWFM, which emits an optical wave and detects changes in the wave, can visualize the structures and behaviors of living cells without any labeling or cytotoxicity, indicating that this microscopy will open up new ways of observing and analyzing cells.

## Data Availability

The data presented in this study are available on request from the corresponding author. The data are not publicly available due to proprietary restrictions.

## Supplementary Materials

The following supporting information can be downloaded at https://www.mdpi.com/article/10.3390/bios15080515/s1, Figure S1: Comparison of OPD images obtained using in-line holography and 3D-OWFM; Figure S2: 3D shape and pseudo-3D reconstruction based on OPD images; Figure S3: Nuclear visualization in HaCaT cells using confocal laser scanning microscopy and 3D-OWFM; Video S1: Time-lapse movie of C3H10T1/2 cells.
