# Supplementary material for: Cell Observation and Analysis with a Three-Dimensional Optical Wave Field Microscope
Source: Biosensors (Basel). 2025 Aug 8;15(8):515. doi: 10.3390/bios15080515 (PMC12384444; doi:10.3390/bios15080515)
Supplement: Supplementary file 1 [file biosensors-15-00515-s001.zip › Supplymantal Figure_250609.pdf]

Supplementary Information

Title :
Cell observation and analysis with a three-dimensional optical wave field microscope

Authors :
Shimon Matsumoto[1,#], Shoko Itakura[2,#], Junta Minato[3], Masahiro Hashimoto[2], Shu Obana[3], Mai Kanai[2], Masaki Kobayashi[4,5], Makiya Nishikawa[2], Kosuke Kusamori[3,*]

Affiliations :
[1]Otsuka Electronics, 1-10 Sasagaoka, Minakuchi, Koka, Shiga 528-0061 Japan
[2]Laboratory of Biopharmaceutics, Faculty of Pharmaceutical Sciences, Tokyo University of Science, 6-3-1 Niijuku, Katsushika, Tokyo 125-8585, Japan
[3]Laboratory of Cellular Drug Discovery and Development, Faculty of Pharmaceutical Sciences, Tokyo University of Science, 6-3-1 Niijuku, Katsushika, Tokyo 125-8585, Japan
[4]Department of Food and Nutrition Science, Graduate School of Humanities and Sciences, Ochanomizu University, 2-1-1 Otsuka, Bunkyo, Tokyo 112-8610, Japan
[5]Institute for Human Life Science, Ochanomizu University, 2-1-1 Otsuka, Bunkyo, Tokyo 112-8610, Japan

[#]Contributed equally to this work.

Supplementary Figure S1.

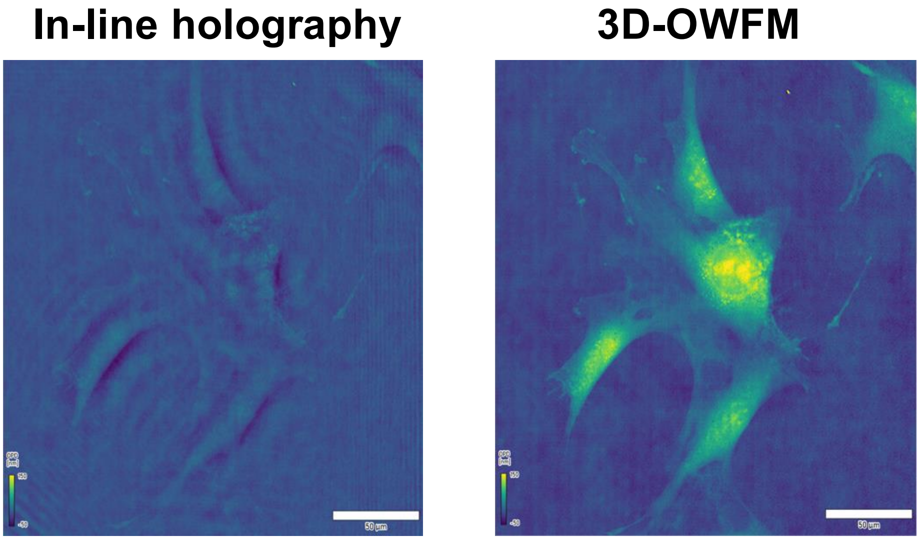

Supplementary Figure S1. Comparison of OPD images of C3H10T1/2 cells obtained using in-line holography and 3D-OWFM. The scale bars indicate 50 μm.

Supplementary Figure S2.

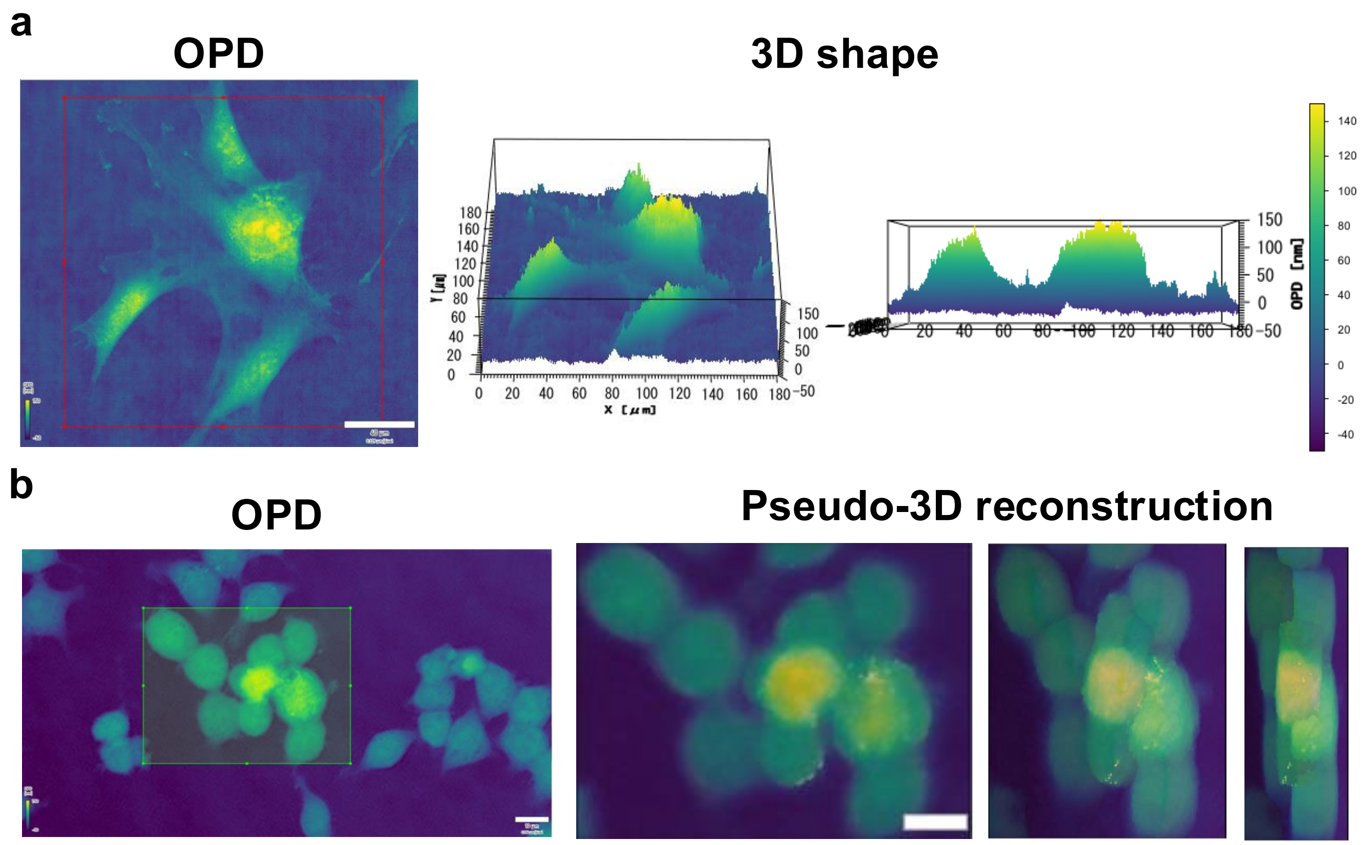

Supplementary Figure S2. (a) OPD image of C3H10T1/2 cells acquired using 3D-OWFM and a 3D shape from a region of interest (ROI) within the image. The 3D view demonstrates the z-directional structure revealed by OPD imaging. The scale bars indicate 40 μm. (b) Pseudo-3D reconstruction of RAW264.7 cells based on z-stacked OPD images obtained from +10 μm to –20 μm relative to the cell surface at 1 μm intervals. The data were extracted from the volumetric dataset acquired within a ±700 μm focal range. The scale bars indicate 10 μm.

Supplementary Figure S3.

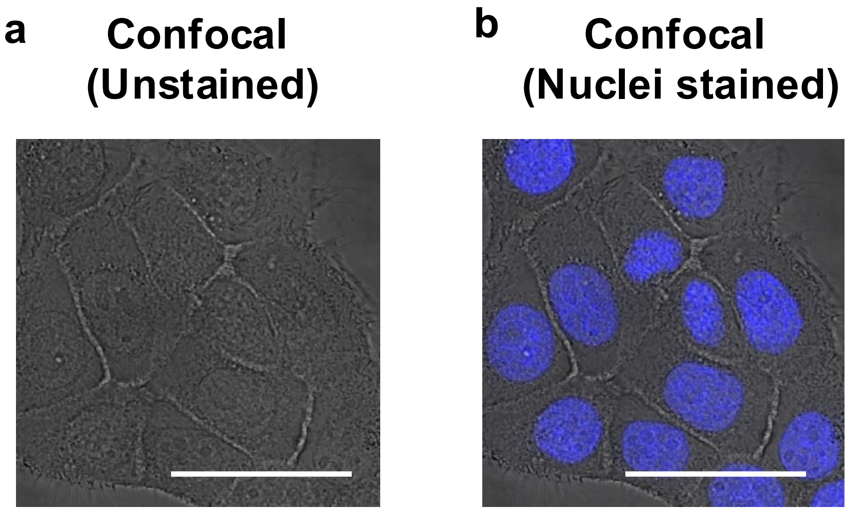

Supplementary Figure S3. Nuclear visualization in HaCaT cells using confocal laser scanning microscopy and 3D-OWFM. Enlarged confocal images of HaCaT cells (a) without nuclear staining, (b) with nuclear staining by Hoechst33342. The scale bars indicate 50 μm.
